# Combined Central and Peripheral Demyelination in a Patient of Multifocal Motor Neuropathy and Positive Anti-myelin Oligodendrocyte Glycoprotein (MOG) Antibodies

**DOI:** 10.7759/cureus.32143

**Published:** 2022-12-02

**Authors:** Alanood E Elterefi, Mahfoud Y Elbashari, Amani Alzaabi, Mohamed E Abouelnaga, Hesham Eissa

**Affiliations:** 1 Internal Medicine, Zayed Military Hospital, Abu Dhabi, ARE; 2 Neurology/Internal Medicine, Zayed Military Hospital, Abu Dhabi, ARE

**Keywords:** demyelinating disorders, multiple sclerosis, chronic inflammatory demyelinating polyneuropathy, multifocal motor neuropathy, combined central and peripheral demyelination, myelin oligodendrocyte glycoprotein (mog) antibodies

## Abstract

Myelin oligodendrocyte glycoprotein (MOG) antibodies have been identified in central nervous system inflammatory demyelinating disorders (MOG antibody disease), inclusive of optic neuritis, transverse myelitis, or acute disseminated encephalomyelitis. The association of MOG antibodies with combined central and peripheral demyelination (CCPD) is not clear. It has been reported in a few cases where MOG antibodies were detected in the serum of patients with chronic inflammatory demyelinating polyneuropathy. However, multifocal motor neuropathy with MOG antibodies is extremely rare. We present a patient who had clinical, neurophysiological, radiological, and biochemical findings that support the diagnosis of CCPD (multifocal motor neuropathy and cord lesion) with MOG antibodies. The patient was treated with a combination therapy of intravenous immunoglobulins plus high-dose methylprednisolone, which resulted in significant improvement.

## Introduction

Combined central and peripheral demyelination (CCPD), although rare, is being increasingly described in case reports. The presentation was previously considered a combination of two diseases that occurred simultaneously [1]. As more cases are reported, it is now a recognized disease spectrum. Patients with CCPD demonstrate neurophysiological findings that are consistent with demyelinating peripheral neuropathy in association with spatial dissemination of demyelination lesions on MRI brain or spinal cord that resemble those of multiple sclerosis (MS) or neuromyelitis optica (NMO) [2]. In almost two-thirds of cases, the nerve conduction study (NCS) was consistent with chronic inflammatory demyelinating peripheral neuropathy (CIDP) [2]. However, any other form of demyelinating peripheral neuropathy may exist. Myelin oligodendrocyte glycoprotein (MOG) antibodies have been reported in multiple cases of CCPD [3-5]. However, it is not clear whether MOG is a serological marker of the disease or CCPD in these cases is part of the MOG antibody disease spectrum with peripheral nervous system involvement.

## Case presentation

**Figure 1:**
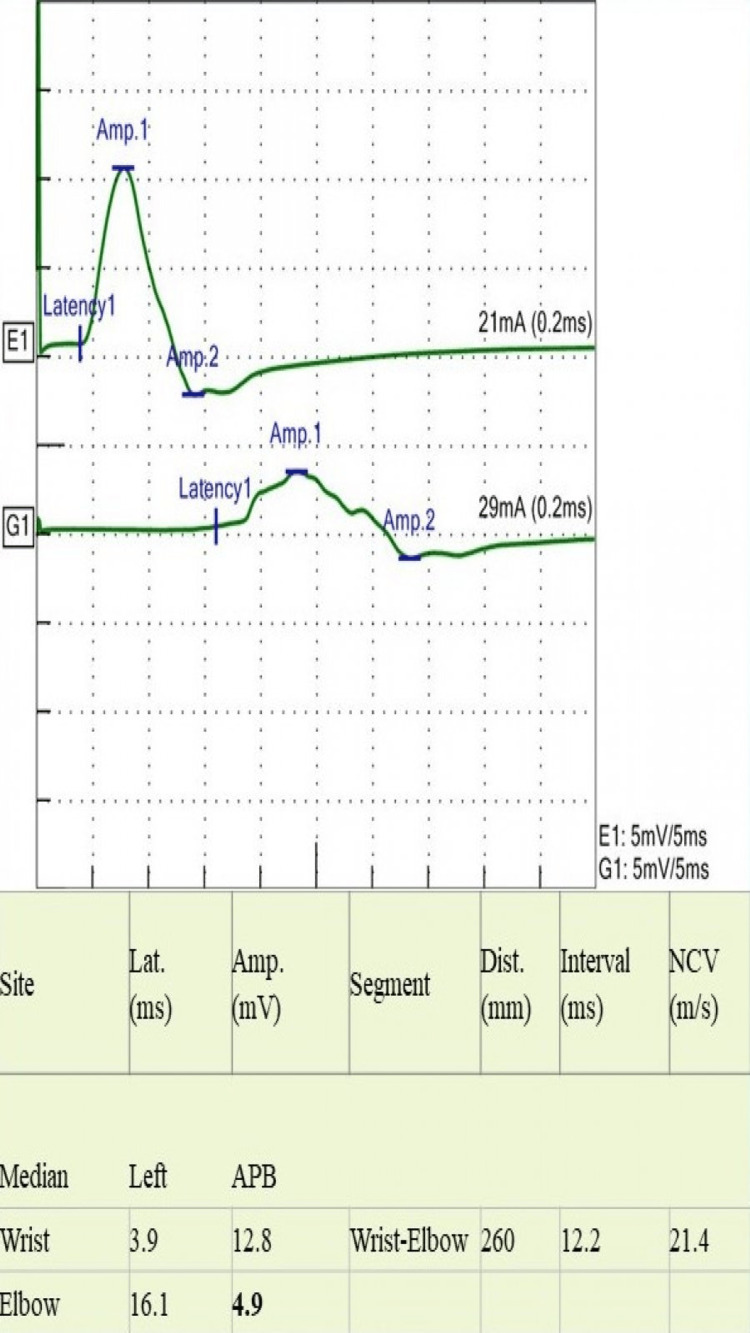
Motor nerve conduction study of median nerve shows conduction block with temporal dispersion in the second site and marked slowing of conduction velocity Lat: latency; Amp: amplitude, Dist: distance; NCV: nerve conduction velocity; APB: abductor pollicis brevis muscle; ms: milliseconds; mV: millivolts; mm: millimeters; m/s: meter per second; mA: milliampere

**Figure 2:**
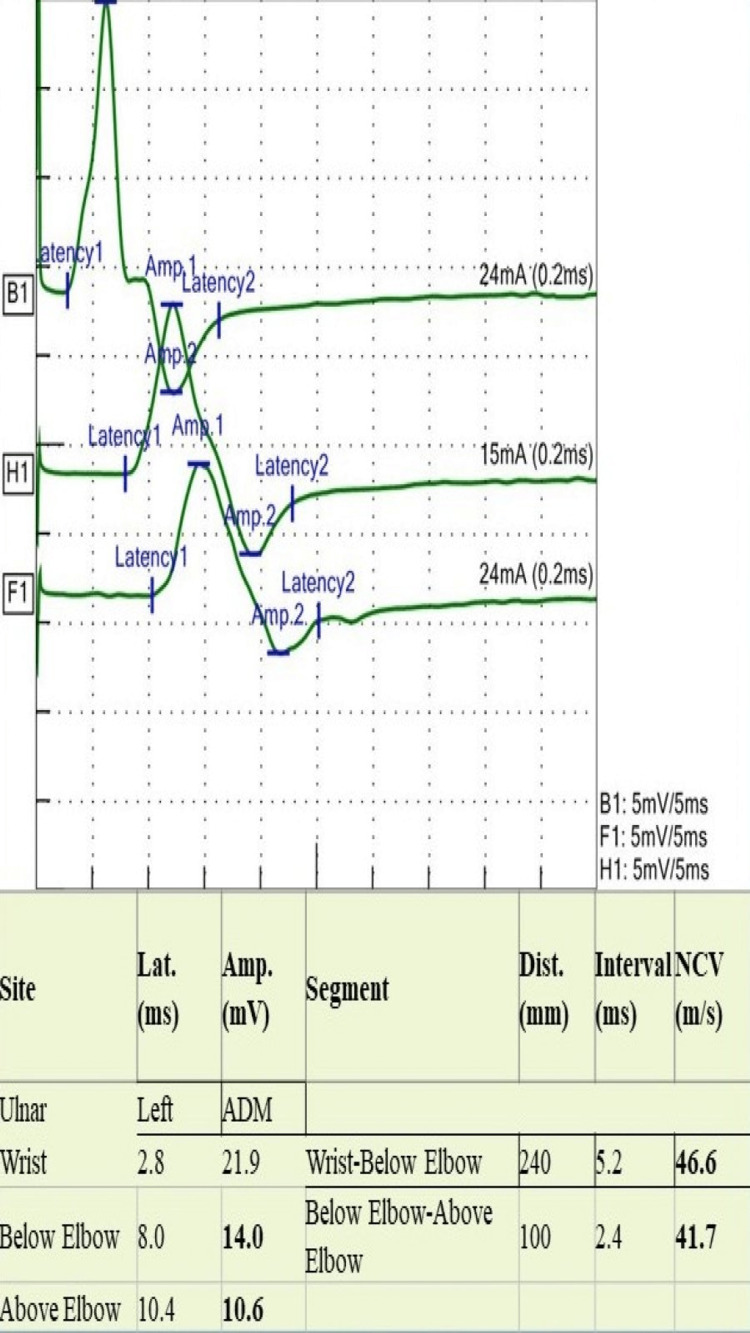
Motor nerve conduction study of ulnar nerve shows conduction block with mild slowing of conduction velocity in the second site and more pronounced in the third site Lat: latency; Amp: amplitude, Dist: distance; NCV: nerve conduction velocity; ADM: abductor digiti minimi muscle; ms: milliseconds; mV: millivolts; mm: millimeters; m/s: meter per second; mA: milliampere

**Figure 3:**
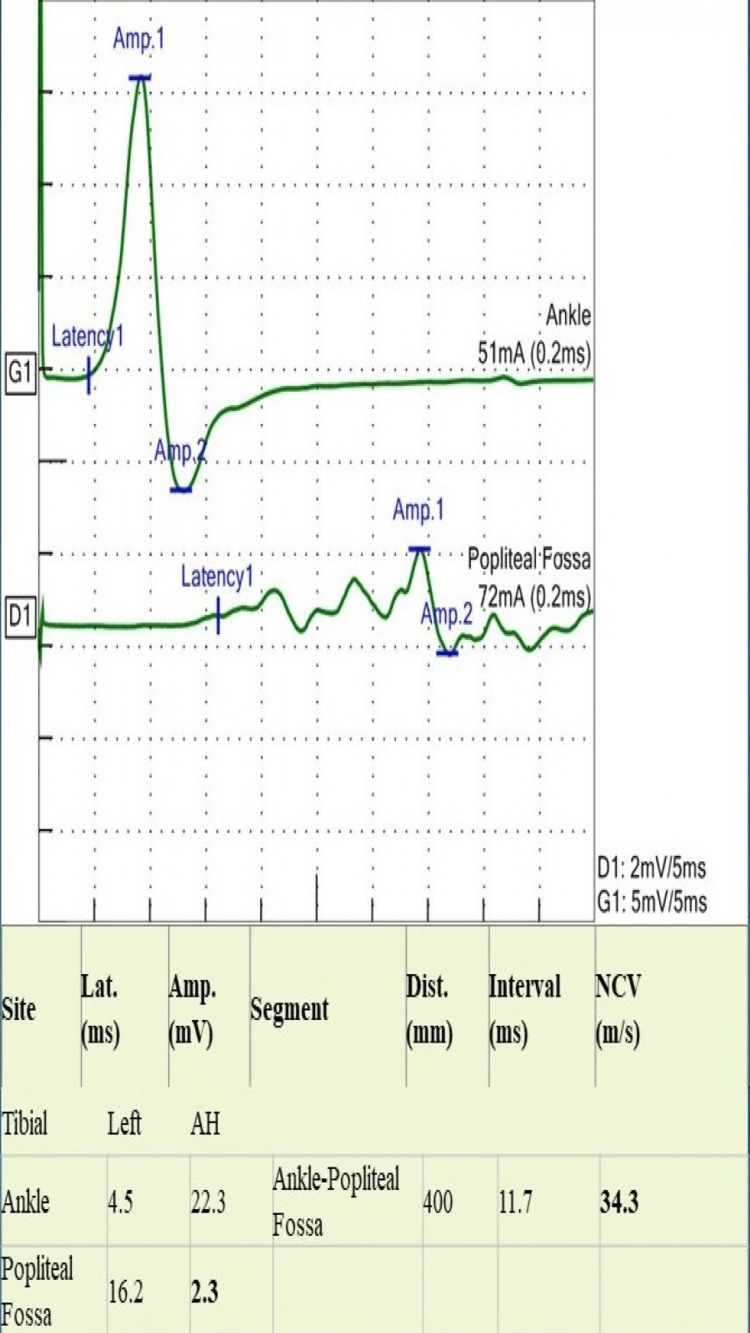
Motor nerve conduction study of the tibial nerve shows marked temporal dispersion in the second site Lat: latency; Amp: amplitude, Dist: distance; NCV: nerve conduction velocity; AH: abductor hallucis muscle; ms: milliseconds; mV: millivolts; mm: millimeters; m/s: meter per second; mA: milliampere

**Figure 4:**
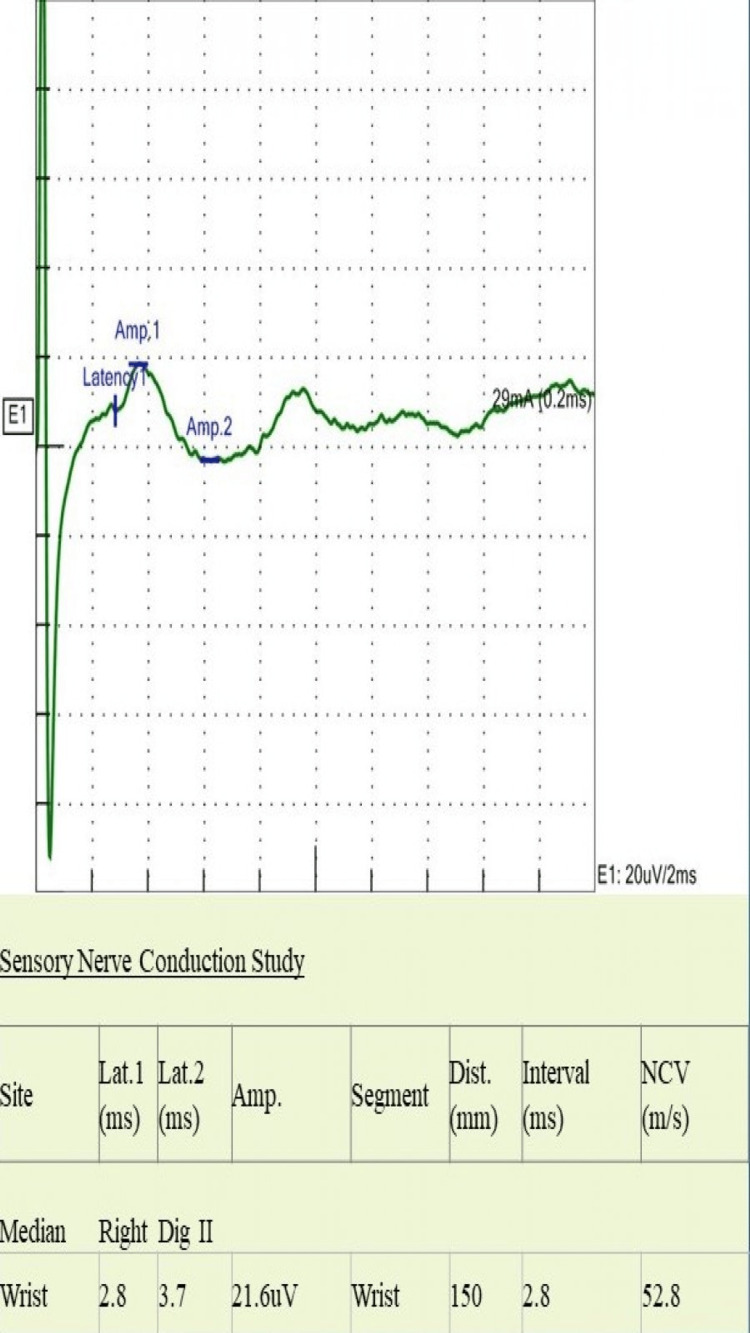
Sensory nerve conduction study of median nerve shows normal latency, SNAP amplitude, and conduction velocity Lat: latency; Amp: amplitude, Dist: distance; NCV: nerve conduction velocity; ms: milliseconds; mV: millivolts; mm: millimeters; m/s: meter per second; mA: milliampere; SNAP: sensory nerve action potentials

**Figure 5:**
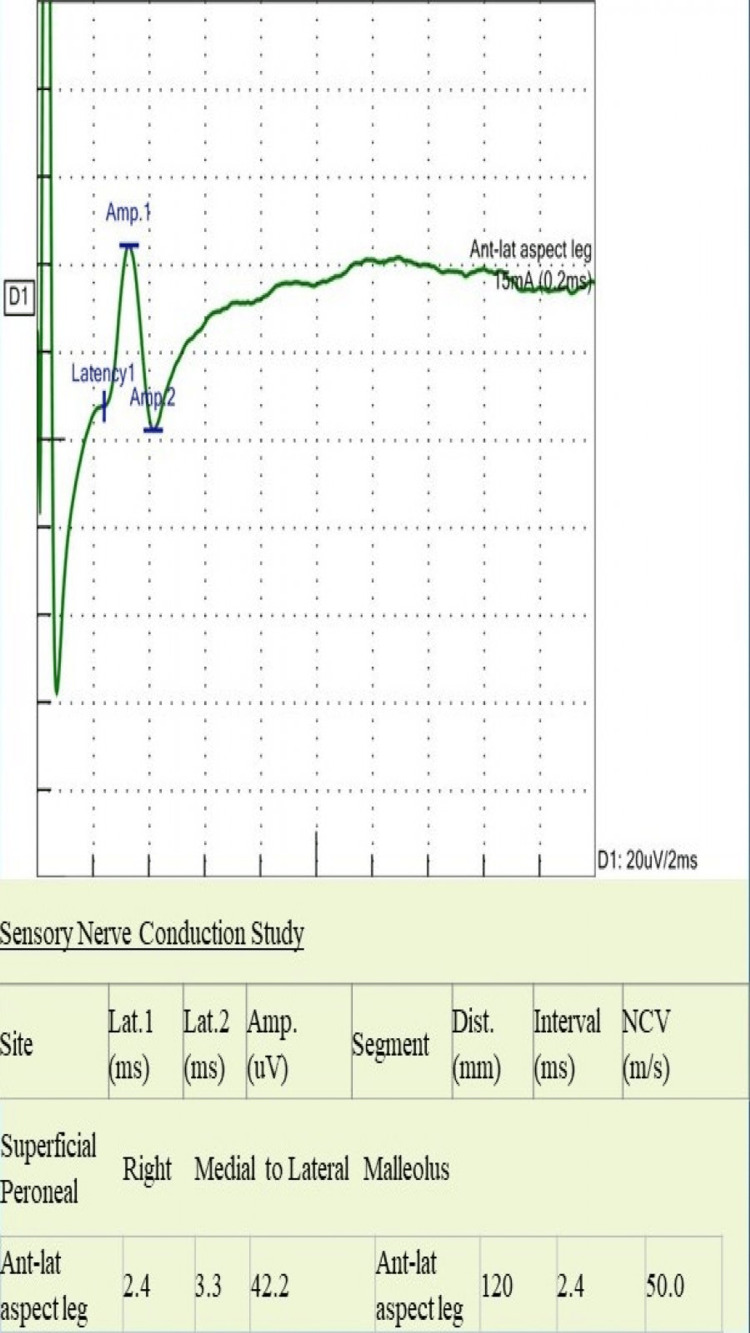
Sensory nerve conduction study of superficial peroneal nerve shows normal latency, SNAP amplitude, and conduction velocity Lat: latency; Amp: amplitude, Dist: distance; NCV: nerve conduction velocity; Ant-lat = anterolateral; ms: milliseconds; mV: millivolts; mm: millimeters; m/s: meter per second; mA: milliampere; SNAP: sensory nerve action potentials

**Figure 6:**
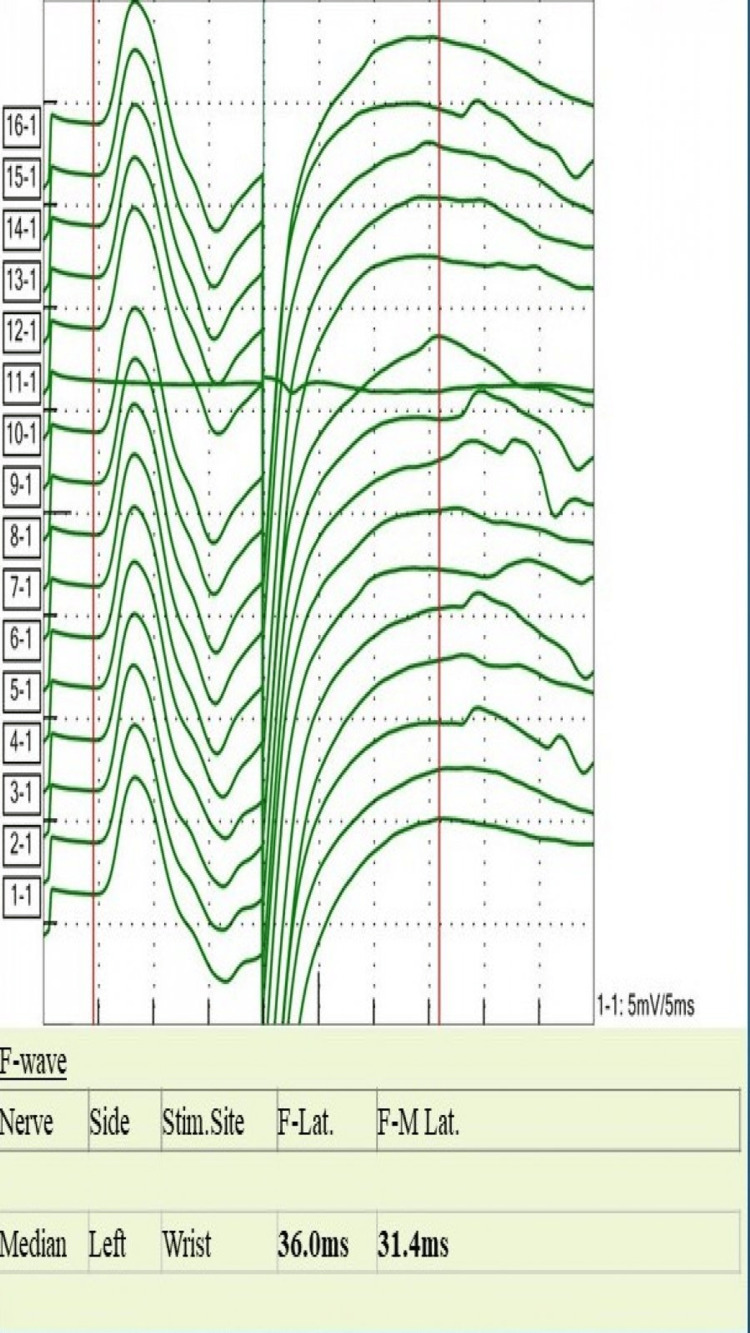
Median nerve F-waves study shows prolonged F-waves Stim. Site: stimulation site; Lat: latency

**Figure 7:**
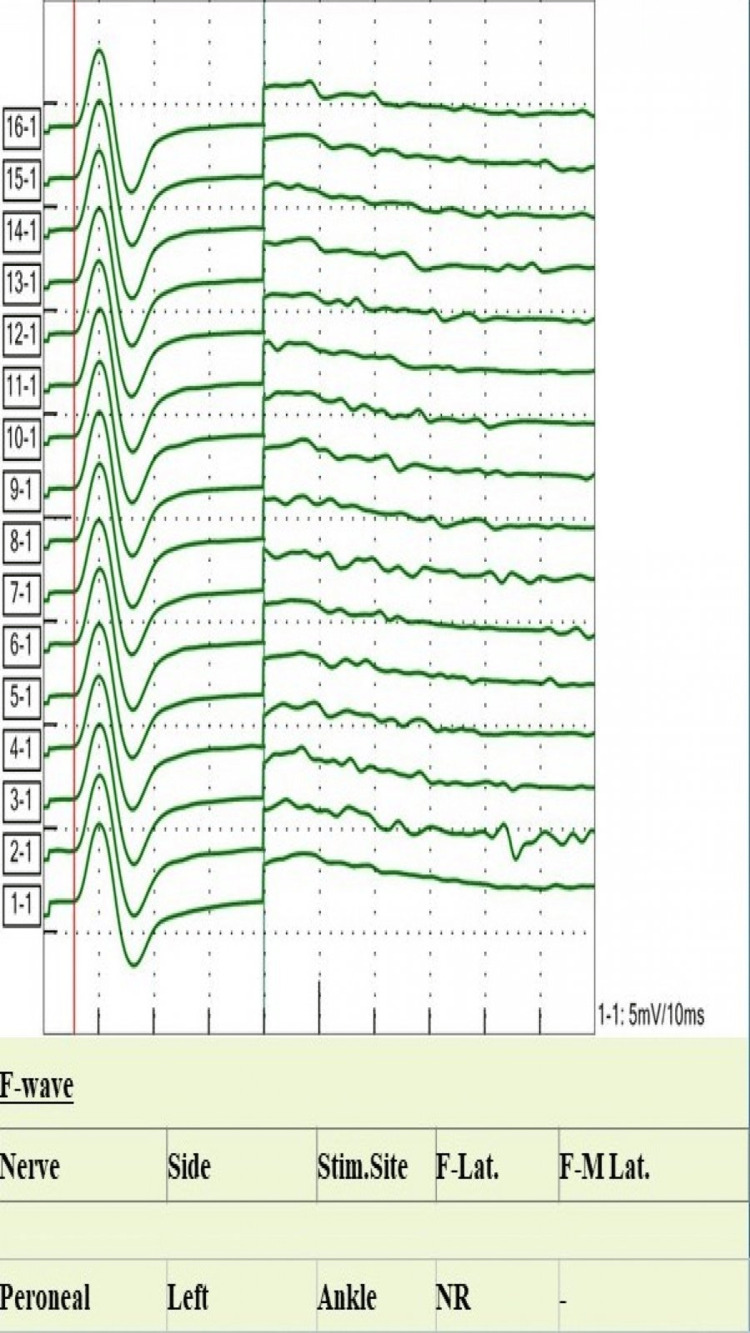
Peroneal nerve F-waves study shows absent F-waves Stim. Site: stimulation site; Lat: latency.

A 24-year-old man presented with a four-month history of asymmetrical numbness and weakness in both upper and lower extremities. The numbness started in his left-hand fingers and soon spread to the entire hand and forearm. It then involved the right thigh, followed by the right foot. Eventually, he had numbness and reduced sensation all over his body. He reported difficulty in walking with a weak hand grip. He had two falls in the bathroom when closing his eyes. He had no visual or facial symptoms. There was no bowel or bladder dysfunction. He had presented to another hospital during his illness, two months prior to his current presentation, where he was admitted with the impression of CIDP and treated with intravenous immune globulin (IVIG). He reported some subjective improvement initially, but he subsequently deteriorated. There was no preceding infection or fever. He had received the coronavirus disease 2019 (COVID-19) vaccine two months after the onset of his symptoms. On examination, he had features of both central and peripheral nervous system involvement; he had bilateral lower motor neuron weakness, more apparent in lower extremities with absent reflexes, and a sensory level at C4 and equivocal plantar responses. Romberg’s sign was strongly positive along with sensory ataxia and pseudo-athetosis. NCS showed normal distal latency, and distal compound muscle action potential (CAMP) with reduced conduction velocity proximally in the median, ulnar, tibial, and peroneal nerves (Figures 1-5). It also revealed features of conduction block with temporal dispersion. F-wave was prolonged or absent (Figures 6, 7). The sensory study was normal. These findings were consistent with multifocal motor neuropathy with conduction block.

**Figure 8:**
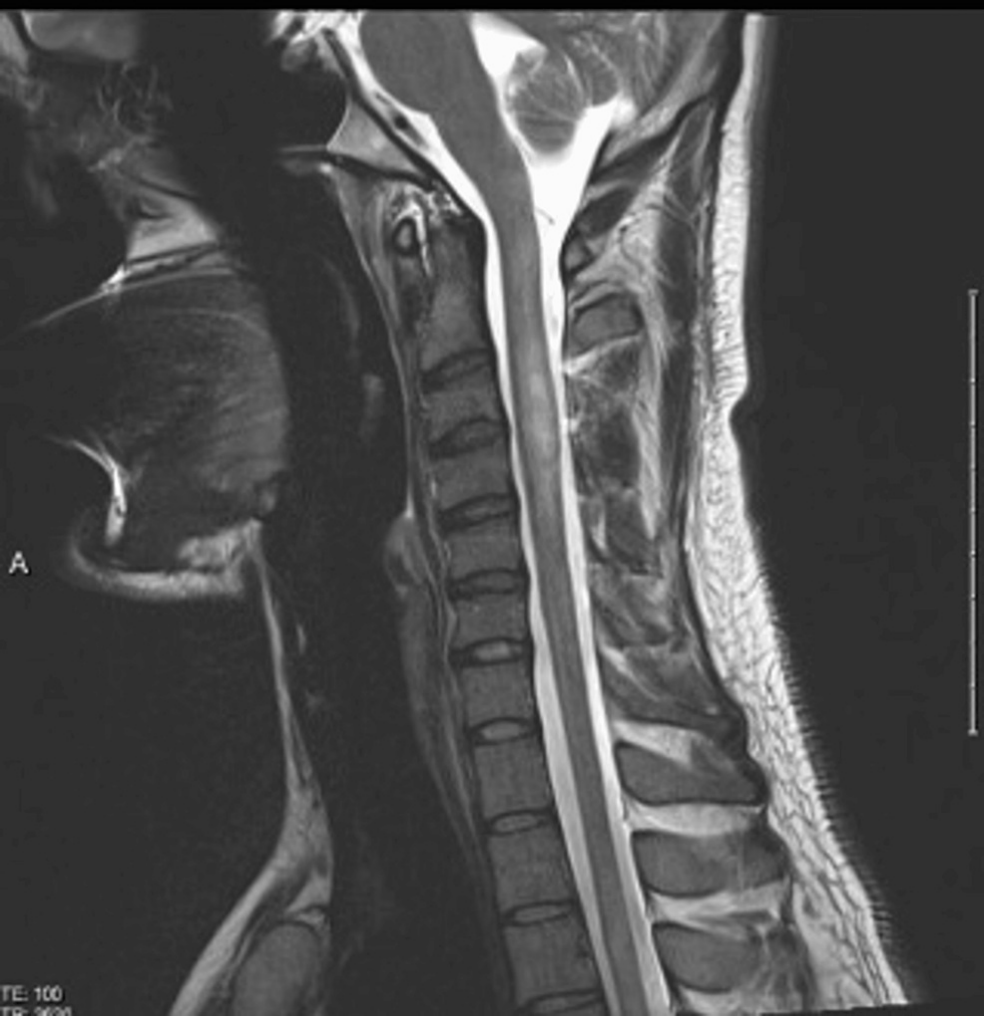
MRI of the cervical spine T2 with contrast. It shows hyperintense signal intensity on T2 image with enhancement post gadolinium.

**Figure 9:**
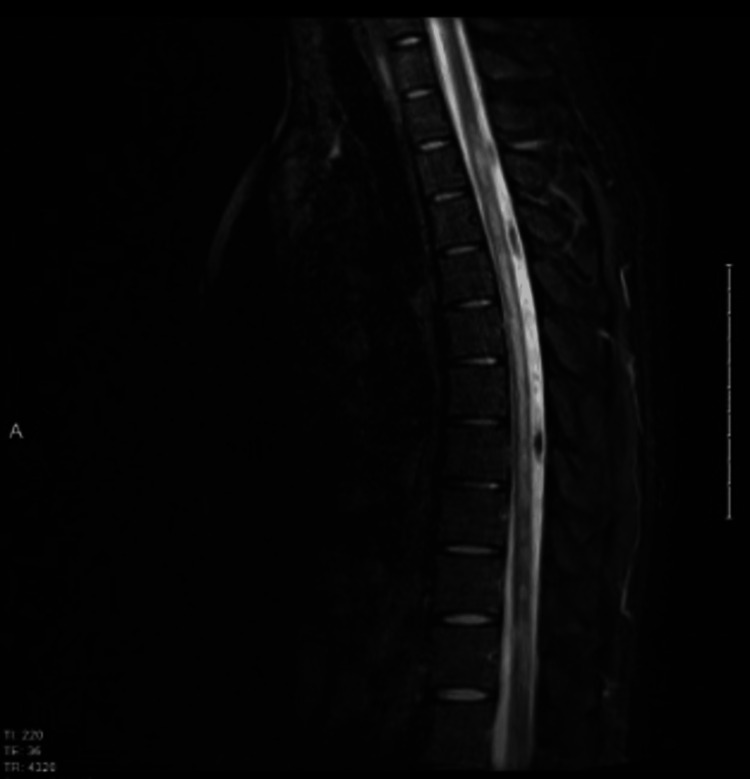
MRI of the thoracic spine T2 with contrast. It shows hyperintense signal intensity with enhancement post gadolinium

Brain MRI was normal. MRI of the spine revealed a contrast-enhancing T2 high signal intensity lesion in the cervical spine involving three segments (Figures 8, 9). 

**Figure 10:**
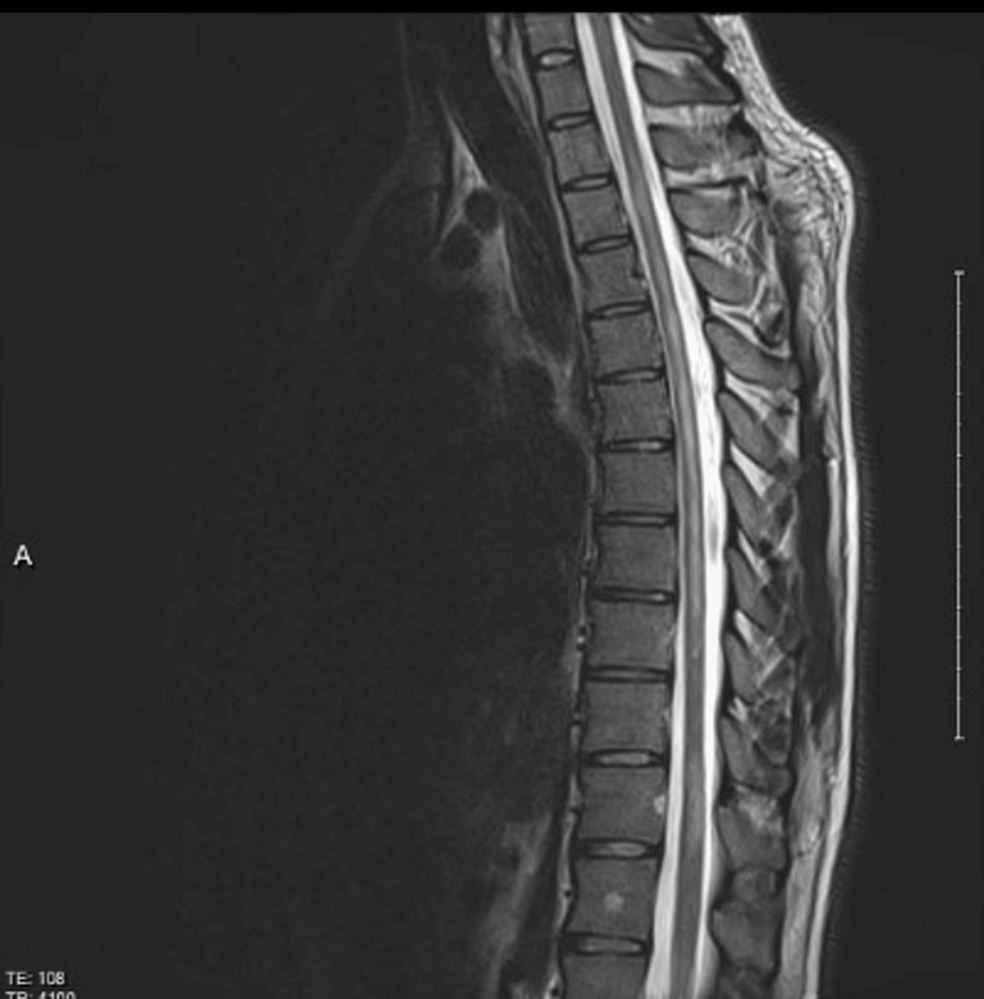
Follow-up MRI of the thoracic spine with contrast (two weeks after the initial MRI)

Cerebrospinal fluid (CSF) protein was 107 mg/dl, CSF WBC was 7 cells/mm^3^ (100% lymphocytes), CSF oligoclonal bands were positive (serum oligoclonal bands were negative), CSF cytology was negative for malignant cells. Aquaporin-4 antibodies were negative both in serum and CSF. Gangliosides profile included anti-GM1 (IgG and IgM) antibodies, anti-GM2 (IgG and IgM) antibodies, anti-GD1a (IgG and IgM) antibodies, anti-GD1b ( IgG and IgM) antibodies, anti-GQ1b (IgG and IgM) antibodies were negative. MOG IgG1 was initially negative. On repeat testing, IgG1 was detected in the patient’s serum (four weeks interval) Using a cell-based assay (indirect fluorescence assay). An extensive workup was done to exclude other toxic metabolic and infectious causes. Rheumatoid factors, antinuclear antibody, serum protein electrophoresis, urinary lead, urinary methylmercury, and urinary thallium all were negative. Angiotensin-converting enzyme, vitamin B1, methylmalonic acid, homocysteine, and IgG4 all were normal. *Brucella abortus *antibodies, *Brucella militances* antibodies, QuantiFERON-TB Gold Plus test (QIAGEN, Hilden, Germany), *Bartonella henselae* antibodies (IgG and IgM), *Mycoplasma pneumoniae* antibodies (IgG and IgM), HIV antigen/antibodies, hepatitis C antibodies, hepatitis B surface antigen, monospot test, dengue virus antibodies (IgG and IgM), mumps virus antibodies (IgG and IgM), and cytomegalovirus (CMV) antibodies (IgG and IgM) were all negative. The visual evoked potential was normal. The patient was diagnosed with MOG antibody-positive CCPD with multifocal motor neuropathy. He received IVIG for five days followed by pulsed steroid therapy for five days. The treatment resulted in significant clinical improvement, despite mild residual deficits. A follow-up MRI of the spine, two weeks later, showed significant improvement in the cervical and thoracic spine lesion (Figure 10). 

The patient came for follow-up 10 days later in the outpatient clinic with a complete improvement of motor power in upper and lower limbs, no more gait difficulty, improvement of sensory symptoms, minimal pseudo athetosis of upper limbs, and deep tendon reflexes were depressed.

## Discussion

CCPD presents as a combination of CNS demyelination lesions, such as optic neuritis and transverse myelitis, and demyelinating peripheral neuropathy. CCPD can present as myeloradiculoneuropathy, encephalopathy, cranial neuropathy, length-dependent peripheral neuropathy, or pseudo-Guillain-Barré syndrome [2,3]. There are no clear diagnostic criteria though. Some proposed the combination of T2 high signal intensity lesions in the brain, optic nerves, or spinal cord on MRI, abnormalities in visual evoked potential, conduction delay, conduction block, temporal dispersion, or F-wave abnormalities suggesting demyelinating neuropathy based on NCS with the exclusion of secondary demyelination, as a reasonable approach [6]. NCS findings may resemble CIDP in two-thirds of patients. MRI findings may be typical of those of MS in almost half of the patients [2].

Serological markers may add great value if present. We propose MOG IgG1 as a potential marker that may be associated with CCPD. MOG IgG1 may also aid in predicting prognosis for these patients, as in MOG encephalomyelitis where persistent seropositivity is associated with an increased chance of relapse when compared with transient seropositivity (patients who are MOG IgG1 negative on follow-up) [7]. MOG antibodies-positive patients are also thought to be at lower risk of further relapses than aquaporin-4-positive patients [8]. 

MOG antibodies are known to be associated with CNS demyelinating disorders. MOG is found on CNS surface myelin sheath and oligodendrocytes processes [9]. Peripheral nervous system involvement in MOG-antibody-related demyelinating diseases is, therefore, poorly understood. Three hypotheses were proposed to explain it. First, MOG in CSF may trigger autoimmunity by mimicry with peripheral myelin proteins. In addition, peripheral expression of MOG was identified in rats and primates [10,4]. Similar findings in humans may explain the associated peripheral nervous system involvement. Individuals with increased susceptibility to autoimmunity are prone to autoimmune attacks that could potentially target nonshared antigens in both central and peripheral nervous system compartments [4].

In our case, the patient’s NCS was consistent with multifocal motor neuropathy with conduction block. The presence of concomitant transverse myelitis on MRI suggested the diagnosis of CCPD. He showed seroconversion as MOG antibodies were initially negative, but became positive on repeat testing.

Multiple treatment options are available with variable outcomes. Corticosteroids, IVIG, and plasmapheresis had different results in different studies. However, the combination of IVIG and pulsed steroid therapy has persistently been superior to either modality alone [11]. The course of MOG antibody-associated CCPD may mimic other MOG antibody-associated disorders, where residual disability develops in 50-80% of patients, with transverse myelitis at onset being the most significant predictor of long-term outcome [12].

## Conclusions

CCPD is uncommon. Furthermore, the combination of MOG antibodies and multifocal motor neuropathy is extremely rare. MOG antibodies may be transiently positive. This seroconversion may be a prognostic indicator of the risk of further relapses and disease outcomes. Combination treatment with IVIG and pulsed steroid therapy is superior to either by itself and results in significant recovery. CCPD has to be acknowledged as a distinct entity and a disease spectrum rather than a combination of two diseases. 
